# Significant elevation of aqueous endothelin-1 in central retinal vein occlusion

**DOI:** 10.1371/journal.pone.0252530

**Published:** 2021-06-02

**Authors:** Hae Min Kang, Md. Hasanuzzaman, So Won Kim, Hyoung Jun Koh, Sung Chul Lee

**Affiliations:** 1 Department of Ophthalmology, Catholic Kwandong University College of Medicine, International St. Mary’s Hospital, Incheon, Republic of Korea; 2 Department of Pharmacology, Asan Medical Center, University of Ulsan College of Medicine, Seoul, Republic of Korea; 3 Department of Ophthalmology, Yonsei University College of Medicine, Seoul, Republic of Korea; 4 Department of Ophthalmology, Konyang University Hospital, Daejeon, Republic of Korea; Massachusetts Eye & Ear Infirmary, Harvard Medical School, UNITED STATES

## Abstract

**Purpose:**

To investigate aqueous humor concentrations of endothelin-1 (ET-1) in patients with central retinal vein occlusion (CRVO) compared with patients with branch retinal vein occlusion (BRVO) and a normal control group.

**Methods:**

A total 80 subjects were included in this prospective study, including 15 patients with CRVO, 20 patients with BRVO, and 45 patients who underwent cataract surgery and had no concomitant ocular disease. Aqueous humor levels of ET-1 were obtained before intravitreal bevacizumab injection (IVB) and after 1 month.

**Results:**

At baseline, the mean aqueous ET-1 level was 12.7±3.6 pg/mL in the CRVO group, 8.0±2.3 pg/mL in the BRVO group, and 2.0±0.9 pg/mL in the control group (P<0.001). After IVB, the mean aqueous level of ET-1 was 3.4±1.9 pg/mL (0.5–6.9 pg/mL) in the CRVO group and 1.8±1.0 pg/mL (0.3–3.2 pg/mL) in the BRVO group (P = 0.008). The mean aqueous ET-1 level was significantly reduced in both the patients with CRVO and those with BRVO (P<0.001).

**Conclusion:**

The mean aqueous humor ET-1 level was significant higher in the patients with CRVO than those with BRVO and in the control group. After IVB, the mean level was significantly reduced in both the patients with CRVO and those with BRVO.

## Introduction

Endothelin-1 (ET-1) is a potent vasoconstrictor peptide that is mainly produced by vascular endothelial cells under physiological conditions [1–3]. ET-1 is essential for maintenance of cardiovascular homeostasis and seems to have an important role in the pathogenesis of vasospasm and hypertension [3–5]. Inside the eye, ET-1 is synthesized and released from the ciliary process [6] and is a potent endogenous vasoconstrictor involved in modulation of ocular blood flow [5, 7]. Inside the eye, ET-1 seems to regulate the blood-retinal barrier (BRB), stimulate growth and migration of cells, and regulate axoplasmic transport. In addition to the vasoconstrictive effect, ET-1 is a humoral factor modulating the blood-brain barrier (BBB) [8]. Eyes with disturbed BBB function can have an increase in ET-1 concentrations around the peripapillary retinal vessels, which leads to some degree of vasoconstriction in glaucoma [8–10].

Retinal vein occlusion (RVO) is one of the most common retinal vascular diseases that can lead to visual loss. Its estimated 15-year cumulative incidence is 2.3% in the population, most cases being branch retinal vein occlusion (BRVO) [11, 12]. Although the exact pathogenesis of RVO remains under investigation, some investigators also focused on the role of ET-1 in RVO pathogenesis. Some experimental studies found that intravitreally or intraconjunctivally injected ET-1 can induce vasoconstriction, and even complete obstruction, of retinal vessels [13–16]. Aqueous humor ET-1 levels and plasma ET-1 levels are elevated in patients with BRVO [17–19]. These study findings suggest the possible role of ET-1 in BRVO pathogenesis. However, no studies of aqueous ET-1 levels in patients with CRVO have been performed.

In this study, we investigated aqueous humor ET-1 levels in patients with CRVO who underwent intravitreal bevacizumab injection (IVB) for macular edema (ME). We then compared these patients with patients with BRVO and with a control group. We also compared aqueous humor ET-1 levels 1 month after IVB in the patients with CRVO and in those with BRVO.

## Methods

### Enrollment of study subjects

This prospective, interventional study was performed at the Catholic Kwandong University College of Medicine, International St. Mary’s Hospital. The study design and protocol were approved by the Institutional Review Board of International St. Mary’s Hospital, Catholic Kwandong University College of Medicine (IS19TISI0006) and adhered to the tenets of the Declaration of Helsinki. A written informed consent approved by the Institutional Review Board of International St. Mary’s Hospital was obtained from each participant at the time of study enrollment.

Study subjects were chosen as participants from December 2018 to March 2020. The study population was classified into three groups. All the participants were treatment-naïve at the time of enrollment, who were diagnosed as unilateral CRVO, unilateral BRVO, or simple cataract during the study period. The inclusion criteria included treatment-naïve patients who were first diagnosed as CRVO, BRVO, or cataract. Excluded from this study were those with concomitant ocular diseases such as diabetic retinopathy, age-related macular degeneration, or glaucoma. In addition, complicated cataracts such as brunscent cataracts, secondary cataracts associated with uveitis or other ocular diseases, phacomophic, or phacolytic cataracts were excluded to minimize possible changes in aqueous humor characteristics.

Glaucoma was defined as evidence of: 1) a glaucomatous visual field defect, confirmed by two reliable visual field tests; 2) glaucomatous optic nerve head (ONH), indicated by a cup-disc ratio > 0.7 and a cup-disc ratio asymmetry >0.2 with diffuse or focal neuroretinal rim thinning, disc hemorrhage, or vertical elongation of the optic cup, or some combination of these clinical signs.

After fulling the inclusion and exclusion criteria, the patients with unilateral CRVO were classified as Group 1 (CRVO group), those with unilateral BRVO were classified as Group 2 (BRVO group), and those who underwent uncomplicated cataract surgery, and had no underlying ocular disease were classified as Group 3 (Control group). If both eyes underwent cataract surgery, the right eye was chosen for analysis.

The primary outcome measure was aqueous ET-1 level in the patients with CRVO, BRVO, and in the control group. Aqueous levels of ET-1 were compared before and after intravitreal anti-vascular endothelial growth factor (VEGF) injections in the patients with CRVO and those with BRVO.

### Ocular examination

An ophthalmologic examination that included a slit-lamp examination, an intraocular pressure measurement using a non-contact tonometer, and a fundus examination after pupillary dilation was performed at each visit. The refractive error of each eye was measured using an autorefractor, and then converted to spherical equivalents [diopters (D)]. Best-corrected visual acuity (BCVA) of the affected eye(s) was checked using a decimal visual acuity chart; each value was converted to a logarithm of the minimum angle of resolution (logMAR). Spectral domain optical coherence tomography (SD OCT) (Spectralis; Heidelberg Engineering, Heidelberg, Germany) imaging were performed using enhanced depth imaging. Fluorescein angiography was performed at the first visit using the Heidelberg Retina Angiograph system (HRA-2; Heidelberg Engineering) with a confocal scanning laser ophthalmoscope.

Central macular thickness (CMT) measured using SD OCT was defined as the mean retinal thickness in the central subfield, a region with a diameter of 1.0 mm around the fovea. The inner and the outer rings had diameters of 3.0 mm and 6.0 mm, respectively. The CMT in the Early Treatment Diabetic Retinopathy Study (ETDRS) macular grid subfields was automatically calculated using the Heidelberg software. The ETDRS grid was manually centered at the fovea if needed. All SD OCT images were reviewed for segmentation errors.

### Sampling of aqueous humor in study subjects

All patients with BRVO or CRVO underwent IVBs for ME. Those who underwent uncomplicated cataract surgeries did not receive IVBs. Each IVB was performed by one retinal specialist (HM Kang) using a standardized approach. Under sterile operating room conditions, anti-VEGF agent (Avastin, Genetech Inc., San Francisco, CA, USA) was drawn into 1.0 ml tuberculin syringes with an attached 19-gauge filter needle. Then, a 30-gauge needle was applied for the intravitreal injection. After topical anesthesia was achieved using 0.5% proparacaine hydrochloride (Alcain^®^; Alcon Inc., Fort Worth, TX, USA), periocular sterilization was done using a 10% povidone-iodine solution. The surgeon cleaned the patient’s skin around the eye with a solution of povidone-iodine 10%; irrigation was performed using povidone-iodine 5% (Betadine^®^; Alcon, USA). After draping, an eyelid speculum was placed. After further topical anesthesia with 4% lidocaine, the injection point was localized 3.0–3.5 mm from the corneal limbus, through the pars plana. For each intravitreal injection, 1.25 mg/0.05 ml bevacizumab (Avastin; Genetech/Roche, San Francisco, CA) was injected into the vitreous cavity. After each injection, topical moxifloxacin (Vigamox^®^; Alcon, USA) was applied to the injection site using a cotton-tip applicator. The same preoperative sterilization preparation was applied for each patient who underwent cataract surgery. After sterilization, anterior chamber paracentesis was performed to drain 0.1 mL aqueous humor, and then either IVB or cataract surgery was performed. Each aqueous sample obtained at this point was set as ‘baseline’. One month after IVB in the patients with BRVO or CRVO, anterior chamber paracentesis was again performed to obtain aqueous humor under the same preoperative sterilization preparation.

### Endothelin-1 concentration measurement

The aqueous humor samples were immediately stored at -80°C until further analysis. On the day of analysis, stored aqueous humor samples were thawed and centrifuged at 400 g for 5 minutes at 4°C to remove cell contents and cell debris [20]. ET-1 was measured using a sandwich ELISA kit (R&D SYSTEMS, QUANTIKINE ELISA endothelin-1 kit, catalog number DET-100) according to the manufacturer’s protocol. In brief, 75 μL aqueous humor samples were pipetted into 96-well plates pre-coated with a monoclonal antibody specific for human ET-1 and that did not cross-react with other isoforms or species. Same volumes of endothelin-1 standards (concentrations 25 pg/mL, 12.5 pg/mL, 6.25 pg/mL, 3.13 pg/mL, 1.56 pg/mL, 0.78 pg/mL, 0.39 pg/mL, and 0 pg/mL) were also pipetted into 96-well plates to make a standard curve. After a 1-hour incubation at room temperature on a shaker, the wells were emptied and washed four times with wash buffer. The 200 μL endothelin-1 conjugates were added to each well and incubated for 3 hours at room temperature on a shaker. The well wash was repeated (four times) and 200 μL substrate solution (Color Reagents A and Color Reagents B with 1:1 ratio) was added to each well. The well contents were then incubated for 30 minutes at room temperature in dark conditions. The enzyme reaction was terminated by adding 50 μL stop solution to each well. Color intensity was measured using a multi-well plate reader (Bio Tek Instruments, Inc., South Korea). The wavelengths for all samples and standards were corrected by subtracting OD540 values from OD450 values. After plotting the standard curve, the corrected OD450 values for samples were extrapolated on the Y-axis and the concentration in pg/mL was measured on the X-axis.

### Statistical analysis

The data were presented as mean ± standard deviation (range) values. Baseline characteristics included age, sex, refractive error, and BCVA at the initial visit. The medical history, which included diabetes mellitus, hypertension, and cerebrovascular disease, was obtained from the medical chart of each patient. IBM SPSS Statistics software for Windows, version 22.0 (IBM Corp., Somers, NY, USA) was used for the statistical analyses. The Kruskal-Wallis test was used for continuous variables and the chi-square test was used for categorical variables. Repeated-measured analysis of variance was used to compare values for mean BCVA, mean CMT, and mean aqueous ET-1 levels between baseline and one month after IVB. Pearson correlation test was used to investigate the correlation of mean aqueous ET-1 levels with mean BCVA and mean CMT. Mauchly’s test of sphericity and Kolmogorov-Smirnov analyses were used to confirm statistical validity. A P value < 0.05 was considered significant.

## Results

### Baseline characteristics of study population

A total 80 subjects were included in this study, including 15 patients in the Group 1 (CRVO group), 20 patients in the Group 2 (BRVO group), and 45 patients in the Group 3 (Control group). None of the patients in the CRVO group and BRVO group developed any CRVO or BRVO in the unaffected contralateral eyes during 12-month of follow-up period. In addition, none in the Group 3 developed any ocular diseases during the same follow-up period. The mean age was 71.0±11.3 years (53–92 years) in the Group 1, 69.0±12.9 years (37–84 years) in the Group 2, and 69.6±8.9 years in the Group 3 (P = 0.878) at the time of diagnosis. There were 7 (46.7%) male patients in the Group 1, 7 (35.0%) in the Group 2, and 25 (55.6%) in the Group 3 (P = 0.374). We found no significant differences when we compared medical histories among the three groups (Table 1). No patients had cerebrovascular disease. The results for baseline characteristics of the study population at the time of diagnosis are presented in Table 1.

**Table 1:** Baseline characteristics of study population at the time of diagnosis.

	Group 1 (CRVO, N = 15)	Group 2 (BRVO, N = 20)	Group 3 (Control, N = 45)	P value
Age (years)	71.0±11.3	69.0±12.9	69.6±8.9	0.878*
Sex (male)	7 (46.7%)	7 (35.0%)	25 (55.6%)	0.374^†^^at^
Laterality (Right eye)	10 (66.7%)	10 (50.0%)	30 (66.7%)	0.576^†^
Hypertension	7 (46.7%)	9 (45.0%)	19 (42.2%)	0.722^†^
Diabetes mellitus	2 (13.3%)	3 (15.0%)	9 (20.0%)	0.517^†^
Angina	0	1 (5.0%)	3 (6.7%)	0.698^†^
Dyslipidemia	8 (53.3%)	12 (60.0%)	28 (62.2%)	0.791^†^
Atrial fibrillation	1 (6.7%)	2 (10.0%)	0	0.810^†^

^†^Chi-square tests for categorical variables, and

*Kruskal-Wallis tests for continuous variable were performed for the statistical analysis. P < 0.05 was used for statistical significance. Abbreviations: BRVO, branch retinal vein occlusion; CRVO, central retinal vein occlusion.

### Aqueous levels of endothelin-1 in study population

At baseline, the mean aqueous ET-1 level was 12.7±3.6 pg/mL (10.3–19.5 pg/mL) in the Group 1, 8.0±2.3 pg/mL (6.9–12.8 pg/mL) in the Group 2, and 2.0±0.9 pg/mL (1.7–2.3 pg/mL) in the Group 3 (P<0.001).

### Changes in aqueous levels of endothelin-1 after intravitreal anti-vascular endothelial growth factor injection

After comparison of baseline aqueous ET-1 levels at baseline, we compared the changes of ocular parameters and mean aqueous ET-1 levels one month after IVBs in the patients with CRVO and BRVO. At baseline, the mean BCVA was 0.6±0.3 logMAR (0.2–1.4 logMAR) in the CRVO group and 0.6±0.4 logMAR (0–1.4 logMAR) in the BRVO group (P = 0.677). The mean CMT was 564.0±154.4 μm (318.0–906.0 μm) in the CRVO group and 471.1±162.5 μm (270.0–716.0 μm) in the BRVO group (P = 0.110).

One month after IVB in the patients with CRVO or BRVO, the mean BCVA was 0.4±0.4 logMAR (0–1.4 logMAR) in the CRVO group and 0.4±0.4 logMAR (0–1.5 logMAR) in the BRVO group (P = 0.458). The mean CMT was 292.8±30.3 μm (244.0–343.0 μm) in the CRVO group and 325.3±80.9 μm (198.0–508.0 μm) in the BRVO group (P = 0.329). The mean aqueous level of ET-1 was 3.4±1.9 pg/mL (0.5–6.9 pg/mL) in the CRVO group, and 1.8±1.0 pg/mL (0.3–3.2 pg/mL) in the BRVO group (P = 0.008).

After intravitreal anti-VEGF injections, the mean CMT, the mean aqueous ET-1 level, and the mean BCVA were significantly reduced in both the patients with CRVO (P<0.001 for all three variables), and in those with BRVO (P = 0.004, P<0.001, and P = 0.012, respectively).

### Correlation of baseline mean aqueous endothelin-1 levels with mean best-corrected visual acuity and mean central macular thickness

We investigated the correlation of baseline mean ET-1 levels with mean BCVA and mean CMT at 12 months after the first IVBs in the patients with CRVO and those with BRVO. The mean BCVA at 12 months was 0.4±0.3 logMAR (0–1.4 logMAR) in the CRVO group and 0.3±0.6 logMAR (0–1.0 logMAR) in the BRVO group (P = 0.289). The mean CMT was 301.2±41.5 μm (243.0–350.0 μm) in the CRVO group, and 300.8±48.6 μm (200.4–348.0 μm) in the BRVO group (P = 0.790).

In the patients with CRVO, mean aqueous ET-1 levels at baseline was not significantly correlated with mean CMT at baseline (P = 0.395) and mean BCVA at baseline (P = 0.427). Mean aqueous ET-1 levels at baseline was not significantly correlated with mean CMT at 12 months (P = 0.589) and mean BCVA (P = 0.333).

In the patients with BRVO, mean aqueous ET-1 levels at baseline was not significantly correlated with mean CMT at baseline (P = 0.143) and mean BCVA at baseline (P = 0.108). Mean aqueous ET-1 levels at baseline was not significantly correlated with mean CMT at 12 months (P = 0.088) and mean BCVA (P = 0.110).

**Fig 1:**
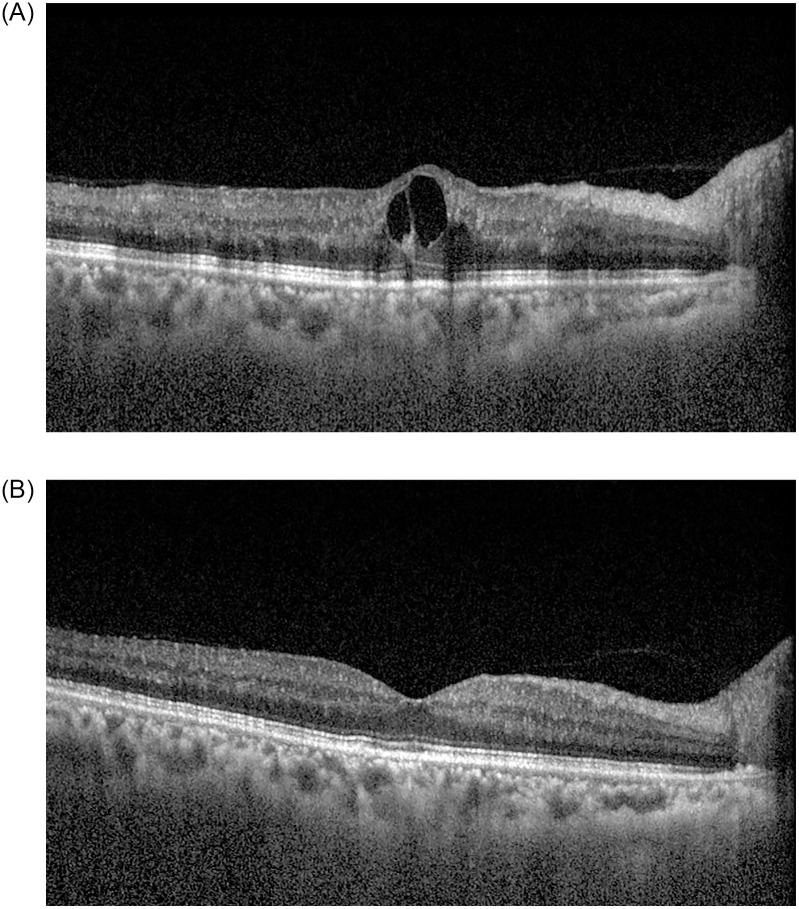
A 63-year-old female patient with central retinal vein occlusion in the left eye. She had no obvious medical history. At the time of diagnosis, best-corrected visual acuity (BCVA) was 0.8 logMAR in the left eye, and diffuse flame-shaped retinal hemorrhages with macular edema was noted in the fundus. Central macular thickness (CMT) was 435.0 μm in the left eye (A). One month after the first intravitreal bevacizumab injection, BCVA in the right eye improved to 0.3 logMAR. The retinal hemorrhages improved, and CMT decreased to 296.0 μm in the left eye (B). The aqueous endothelin-1 level was 12.1 pg/mL at the time of the first intravitreal bevacizumab injection and decreased to 6.9 pg/mL one month after the first injection.

**Fig 2:**
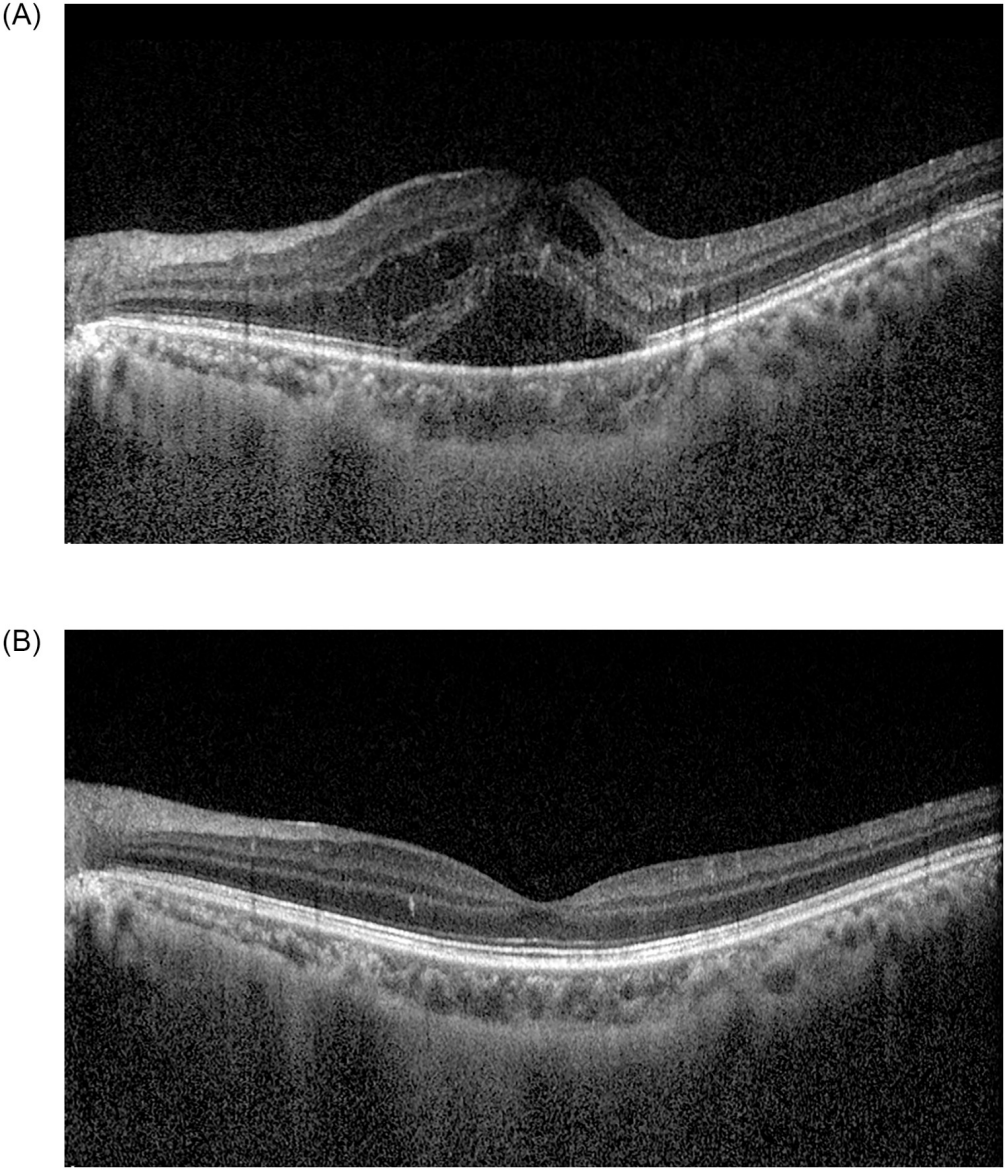
A 40-year-old male patient with branch retinal vein occlusion in the right eye. He had a history of dyslipidemia. At the time of diagnosis, best-corrected visual acuity (BCVA) was 0.3 logMAR in the left eye. A fundus examination revealed flame-shaped retinal hemorrhage and cotton-wool spots along the superotemporal vascular arcade in the left eye. The central macular thickness (CMT) was 586.0 μm in the left eye (A). One month after intravitreal bevacizumab injection, BCVA in the left eye improved to 0.2 logMAR. The retinal hemorrhages improved, and CMT decreased to 286.0 μm in the left eye (B). The aqueous endothelin-1 level was 12.8 pg/mL at the time of the first intravitreal bevacizumab injection, and then decreased to 1.7 pg/mL one month after injection.

Representative cases are shown in Figs 1 and 2.

## Discussion

In this study, we investigated aqueous levels of ET-1 in patients with CRVO. The aqueous ET-1 level in the CRVO group was significantly higher than that in the BRVO group and in the control group. Compared with the control group, the mean aqueous ET-1 level was significantly higher in both the CRVO and BRVO groups. After IVB, the mean aqueous ET-1 level was significantly reduced in both the CRVO and BRVO groups. However, the mean aqueous ET-1 levels were not significantly correlated with mean BCVA and mean CMT at baseline and at 12 months in both the patients with BRVO and those with CRVO.

Mean aqueous ET-1 levels were significantly reduced after intravitreal anti-VEGF injection. The results suggested that an increased ET-1 level can be effectively reduced using an anti-VEGF agent. Study results suggest that a reduction in VEGF leads to a reduction in ET-1; this change can be explained by the stimulatory interaction between VEGF and ET-1 [18]. Then, reduced ET-1 cannot further constrict retinal vessels in patients with RVO.

Plasma ET-1 levels increase in patients with BRVO [17, 19]. One study found that increased ET-1 levels in circulating blood leads to local constriction of retinal veins [21]. The researchers postulated that the resultant constriction of retinal veins further increases retinal venous pressure (RVP) and may contribute to RVO pathogenesis.

The pathophysiology of CRVO remains under investigation. One histopathologic study found a thrombus occluding the lumen of the CRV at or just proximal to the lamina cribrosa [22]. Within the retrolaminar portion of the optic nerve, the central retinal artery (CRA) and CRV are aligned parallel in a common sheath where they are naturally compressed as they cross through the rigid sieve-like openings in the lamina cribrosa [23]. Both the CRA and CRV are subject to compression from mechanical stretching of the lamina, which leads to subsequent impingement on the CRV [23].

There are two blood-tissue barriers in the optic nerve head. The microvessels of the prelaminar part form the inner BBB, and the tissue of Elschnig forms the outer BBB [8]. The peripapillary choriocapillaris are largely fenestrated, allowing free leakage from blood [8]. These two BBBs are incomplete; this configuration can increase vulnerability to noxious, circulating substances [8]. Systemic diseases such as hypertension, diabetes mellitus, and dyslipidemia are well-known risk factors for CRVO, and mild repeated ischemia-reperfusion events in the body may increase plasma levels of ET-1 [24–26]. Because of an incomplete BBB in the ONH and peripapillary choriocapillaris, elevated plasma ET-1 levels can easily increase the local ET-1 concentrations around peripapillary retinal vessels and result in some extent of vasoconstriction, as suggested in patients with glaucoma [8–10]. Elevated local ET-1 may further constrict the CRV and associated collateral vessels at the level of the lamina cribrosa or peripapillary area. The subsequently elevated RVP may lead to progressively decreased perfusion pressure (PP). If PP drops below a critical limit, the hypoxic retina starts to further increase the local ET-1 level. Then, a subsequent vicious cycle includes further reduction of PP and breakdown of the BRB. In turn, retinal hemorrhages and retinal edema develop in CRVO. The elevated aqueous humor ET-1 levels found in our study support this hypothesis in the pathogenesis of CRVO.

In this study, we also found that aqueous humor ET-1 levels were higher in the patients with CRVO than those with BRVO. If an elevated plasma ET-1 level is involved in the pathogenesis, we can assume that the patients who develop CRVO may have much higher plasma ET-1 levels or are more prone to the leakage/transmission of plasma ET-1 through BBBs of the ONH and peripapillary choriocapillaris. A higher local ET-1 level may lead to development of CRVO, and a lower level of local ET-1 may lead to development of BRVO. Because one experiment found that ET-1 can induce dose-dependent vasocontractile responses [13], our hypothesis may explain differences in development of CRVO versus BRVO. However, this study lacked investigation of plasma ET-1 levels in the study population. Further studies should be performed to validate our hypothesis.

If plasma ET-1 levels affect RVO development, there should be another factor that explains unilateral development of RVO. Both CRVO and BRVO usually occur unilaterally, and about 10% of patients with RVO had bilateral disease at baseline [27]. The findings indicated that 4.5% of the study population developed fellow-eye RVO [27], and the unilateral-to-bilateral conversion rate was 1.5% per year [27]. If ET-1 in circulating blood affects both eyes, we should investigate factors affecting the tendency for unilateral RVO development. Anatomical or vascular variation in the ONH or peripapillary vasculature, or both, might affect local concentrations of ET-1 that affect retinal vasoconstriction in each eye. Another hypothesis is that differences in susceptibility in each eye affect this unilateral preference. If, despite RVO development, an elevated plasma ET-1 level persists due to the presence of systemic vascular disease, the less susceptible eye can also develop RVO, as shown by the unilateral-to-bilateral conversion rate. Further studies of the anatomic variation and peripapillary structure in RVO are needed to reveal factors involved with unilateral RVO development.

In our study, we found that aqueous humor ET-1 level was significantly reduced after IVBs in patients with CRVO and in those with BRVO. Hypoxia increases hypoxia-inducible factor-1 alpha, leading to upregulation of genes such as erythropoietin, VEGF, and ET-1 [28]. Reduction in VEGF normally leads to reduction in ET-1; this relationship is explained by the stimulatory interaction between VEGF and ET-1 [29].

We found that mean aqueous ET-1 level at baseline was not significantly correlated with mean BCVA and mean CMT at baseline and at 12 months in both the patients with CRVO and those with BRVO. As our results imply, ET-1 may not directly affect functional and anatomical outcomes, although it seems to be involved in the pathogenesis of CRVO and BRVO. Because disease itself and various complications associated with CRVO or BRVO such as foveal ischemia, macular edema, and vitreous hemorrhage are associated with functional and anatomical outcome in CRVO and BRVO, ET-1 may not directly affect clinical outcomes. However, if ET-1 is involved in the pathogenesis of CRVO and BRVO, prevention or early intervention for these diseases may be beneficial to preserve vision in these patients. Thus, further studies are warranted to investigate the possible impact of ET-1 in the clinical outcome in the patients with CRVO and those with BRVO.

This study has some limitations, including a relatively small number of patients in the study population. Our study lacks aqueous humor sampling of the unaffected fellow eyes and plasma ET-1 levels in the study subjects. Our study also lacks vitreous sampling in the study population. If these data can be analyzed, we may find the exact role of ET-1 in the pathogenesis of CRVO and BRVO, and further understandings for these disease entities can be possible. Based on the current results, we plan to investigate further to determine the exact pathogenesis of RVO and the exact role of ET-1 to prevent further visual loss from this disease entity.

In conclusion, the mean aqueous humor ET-1 level was significantly higher in CRVO than BRVO or normal controls. After IVB, aqueous ET-1 levels in both the CRVO group and the BRVO group were significantly reduced.

## Supporting information

S1 Fig Fundus of a 63-year-old female patient with central retinal vein occlusion in the left eye. Diffuse flame-shaped retinal hemorrhages with macular edema was noted (A). One month after the first intravitreal bevacizumab injection, the retinal hemorrhages improved (B).
(TIF)

S2 Fig Fundus of a 40-year-old male patient with branch retinal vein occlusion in the right eye. A fundus examination revealed flame-shaped retinal hemorrhage and cotton-wool spots along the superotemporal vascular arcade in the left eye (A). One month after intravitreal bevacizumab injection, the retinal hemorrhages improved (B).
(TIF)
