# Supplementary figures and images for: Significant elevation of aqueous endothelin-1 in central retinal vein occlusion
Source: PLoS One. 2021 Jun 2;16(6):e0252530. doi: 10.1371/journal.pone.0252530 (PMC8171894; doi:10.1371/journal.pone.0252530)

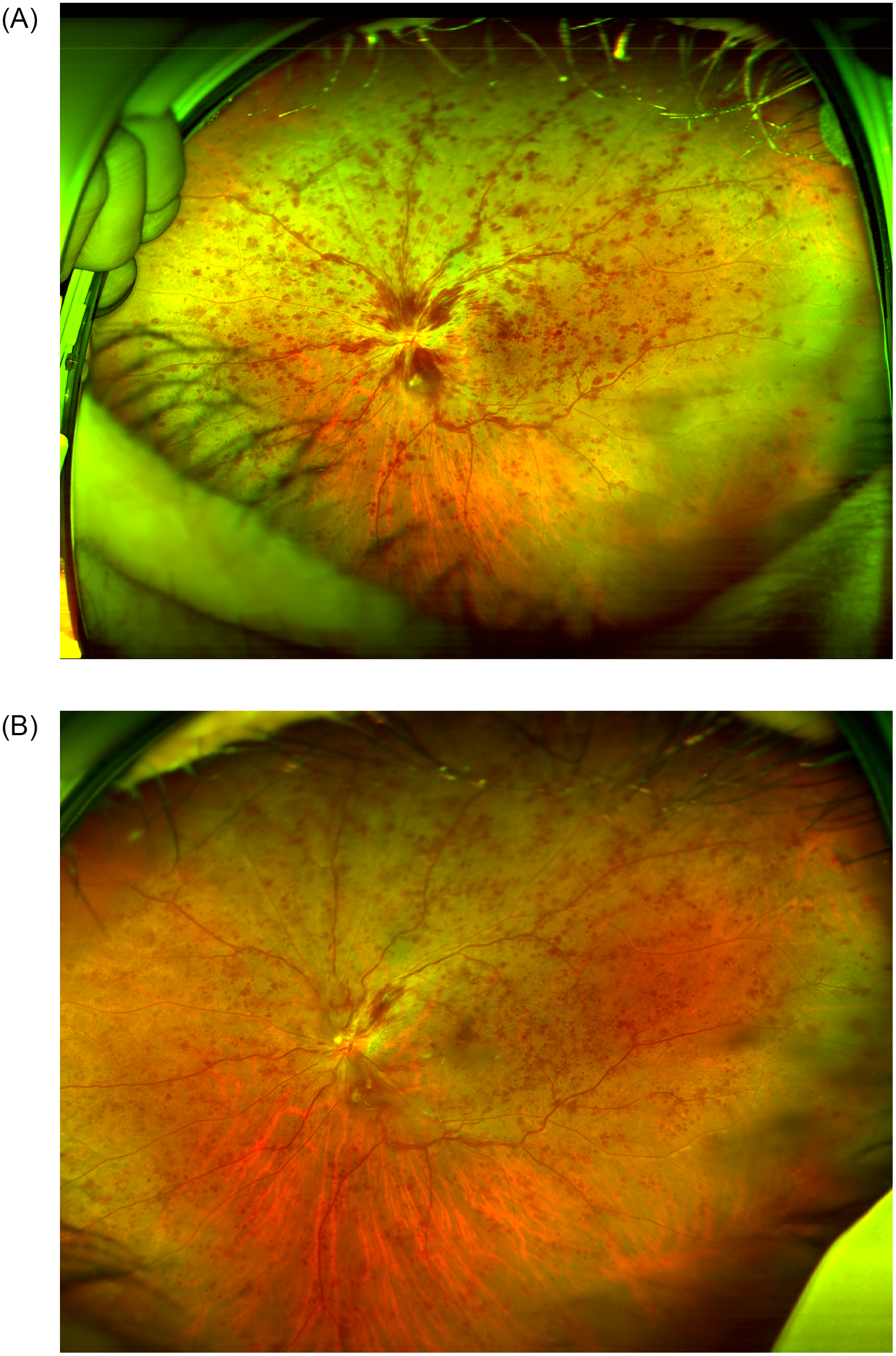

Supplement: S1 Fig — Diffuse flame-shaped retinal hemorrhages with macular edema was noted (A). One month after the first intravitreal bevacizumab injection, the retinal hemorrhages improved (B). (TIF) [file pone.0252530.s001.tif]

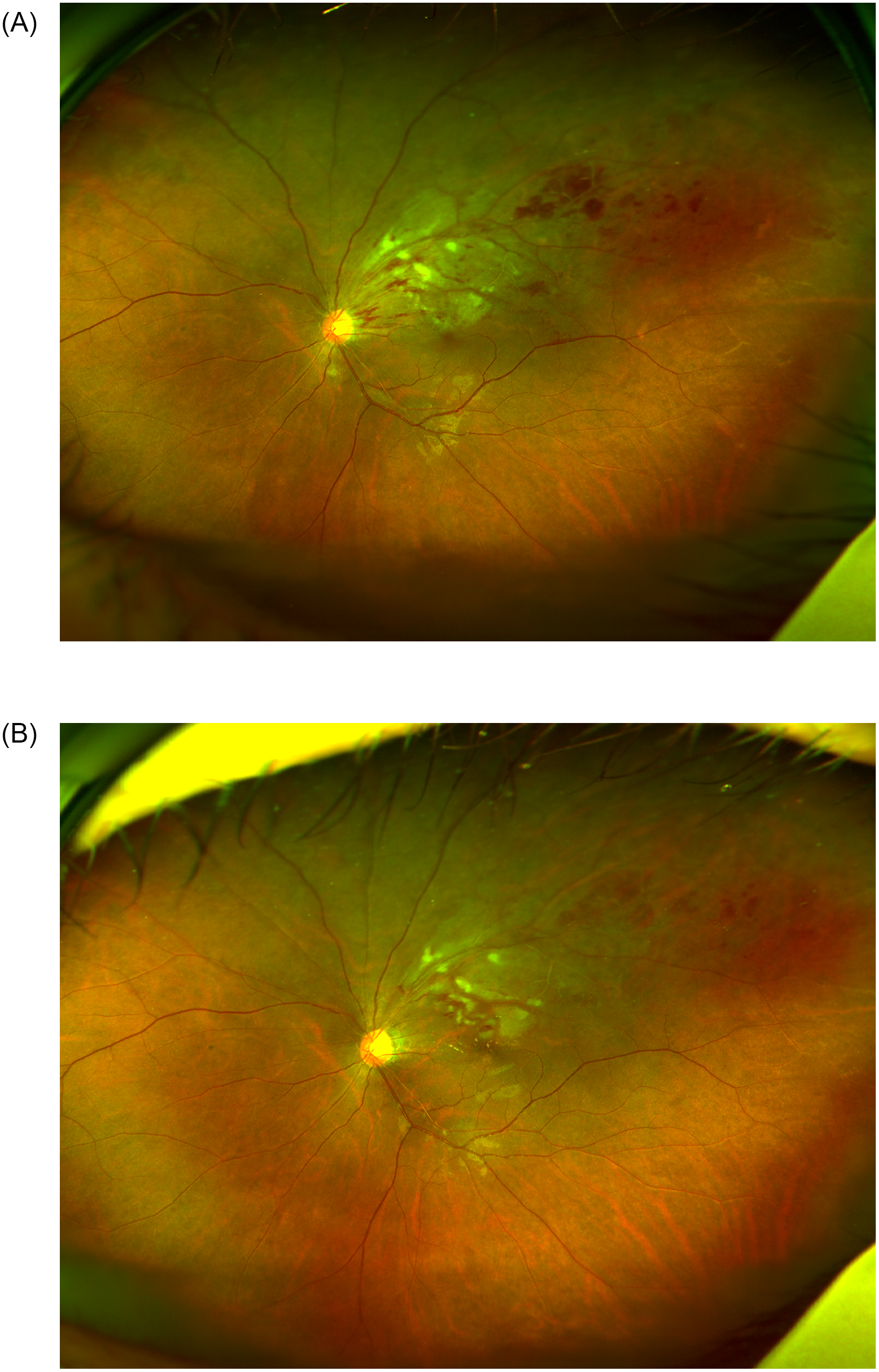

Supplement: S2 Fig — A fundus examination revealed flame-shaped retinal hemorrhage and cotton-wool spots along the superotemporal vascular arcade in the left eye (A). One month after intravitreal bevacizumab injection, the retinal hemorrhages improved (B). (TIF) [file pone.0252530.s002.tif]
